# Synthetic Peptides against Plant Pathogenic Bacteria

**DOI:** 10.3390/microorganisms10091784

**Published:** 2022-09-03

**Authors:** Esther Badosa, Marta Planas, Lidia Feliu, Laura Montesinos, Anna Bonaterra, Emilio Montesinos

**Affiliations:** 1 Laboratory of Plant Pathology, Institute of Food and Agricultural Technology-CIDSAV-Xarta, University of Girona, Campus Montilivi, 17071 Girona, Spain; 2 LIPPSO, Department of Chemistry, University of Girona, Campus Montilivi, 17071 Girona, Spain

**Keywords:** synthetic peptides, pathogenic bacteria, biopesticides, lytic peptides, plant defense

## Abstract

The control of plant diseases caused by bacteria that seriously compromise crop productivity around the world is still one of the most important challenges in food security. Integrated approaches for disease control generally lack plant protection products with high efficacy and low environmental and health adverse effects. Functional peptides, either from natural sources or synthetic, are considered as novel candidates to develop biopesticides. Synthetic peptides can be obtained based on the structure of natural compounds or de novo designed, considering the features of antimicrobial peptides. The advantage of this approach is that analogues can be conveniently prepared, enabling the identification of sequences with improved biological properties. Several peptide libraries have been designed and synthetized, and the best sequences showed strong bactericidal activity against important plant pathogenic bacteria, with a good profile of biodegradability and low toxicity. Among these sequences, there are bacteriolytic or antibiofilm peptides that work against the target bacteria, plant defense elicitor peptides, and multifunctional peptides that display several of these properties. Here, we report the research performed by our groups during the last twenty years, as well as our ongoing work. We also highlight those peptides that can be used as candidates to develop novel biopesticides, and the main challenges and prospects.

## 1. Introduction

Losses in crop yield caused by plant diseases account for between 16 and 18 % of global agricultural productivity [1], which result in a substantial economic impact and endanger food security. Plant diseases caused by bacteria are the main factors limiting crop production. Some especially relevant plant pathogenic bacteria are *Erwinia amylovora*, *Xanthomonas arboricola* pv. vesicatoria and *Pseudomonas syringae* pv. syringae, the causal agents of fire blight in the Rosaceous family, the bacterial leaf spot on tomatoes and peppers and the floral bud necrosis, respectively [2,3]. The current effective approaches to control bacterial diseases are mostly based on the use of copper compounds or antibiotics, with the latter being allowed in several countries, but not in Europe [4,5]. However, their extensive use has adverse effects on the environment and human health, as well as on animal health, and evokes antibiotic resistance of bacterial pathogens [6]. Even so, the difficulty of control increases in the case of fastidious phytopathogenic bacteria, such as *Xylella fastidosa* and *Candidatus* Liberibacter asiaticus, for which there are still no effective methods for disease control in infected plants, due to the lack of appropriate bactericides and the difficulty of accessing the vascular vessels, where these pathogens propagate. Current control is based on preventive measures to limit their spread, such as eradication, pathogen-free propagation of plant material, exclusion, and vector control [7]. Taking the regulatory framework of pesticides (International Plant Protection Convention) into account, there is a strong need for alternative, environmentally compatible products to control phytopathogens. Antimicrobial peptides (AMPs) have been identified as good candidates for plant disease control, and show strong potential to manage diseases caused by plant pathogenic bacteria, fungi and phytoplasmas [8,9]. In fact, the main properties of AMP include the following: (1) high antimicrobial activity with minimal inhibitory concentration (MIC) at the micromolar level, being, in general, similar to that of conventional antibiotics and significantly lower than that of copper compounds, (2) quick response in the target pathogen, (3) higher biodegradability than conventional pesticides, (4) low cytotoxicity, and (5) multiple modes of action [10]. The main mechanism of action of AMPs is the electrostatic interaction with the bacterial membrane, leading to its disruption that results in bacterial death. It has also been described that some AMPs can interact with intracellular targets, such as nucleic acids and proteins [11,12]. Apart from being antibacterial, AMPs can act as pathogen-associated molecular patterns (PAMPs) or plant elicitor peptides (Peps), stimulating the plant immune system [13,14,15].

There is currently an abundance of updated information on antimicrobial peptides of different origins, with numerous recent reviews providing an overview of the progress on natural peptides of plant [16,17,18,19], animal [20,21,22,23] or microbial origin [24], as well as synthetic peptides based on the structure of natural sequences [25]. Research on the applications of these peptides, such as in agriculture [19,26] or human health [25,27,28,29], has also been extensively covered in previous reviews.

During the last twenty years, our research groups from the University of Girona have been extensively working to design and develop AMPs, with the aim to identify potential candidates to combat phytopathogenic bacteria of great economic importance. In particular, we have been interested in finding AMPs that are useful to control diseases caused by *Erwinia amylovora* (Ea) in pears, *Xanthomonas axonopodis* pv. vesicatoria (Xav) in tomatoes and pepper plants, *Xanthomonas fragariae* (Xfr) in strawberries, *Xanthomonas axonopdis* pv. pruni (Xap) in stone fruit, *Pseudomonas syringae* pv. syringae (Pss), pv. tomato (Pst) or pv. actinidiae (Psa) in Rosaceous plants, tomato or kiwi plants, respectively.

More recently, our efforts have been focused on the control of the quarantine pathogens *X. fastidiosa* (Xf), affecting at least 638 plant species [30] and *Candidatus* Liberibacter asiaticus, a fastidious, phloem-limited bacterium that affects citrus [7]. *X. fastidiosa* causes Pierce’s disease in grapevines, almond leaf scorch, citrus variegated chlorosis and olive decay syndrome [31] and *Candidatus* Liberibacter asiaticus is the main causative agent of Huanglongbing (HLB) [32,33]. Within this context, up to now, we have developed linear and cyclic peptides, which have been designed from the structure of natural peptides or based on the common features of AMPs. Our main objective is to find short sequences with an optimal biological profile in terms of high antibacterial activity, low toxicity and high stability to protease degradation.

The aim of this review is to contextualize our research lines developed in the framework of several national and European projects, which have resulted in the identification of good candidates to be used in integrated plant bacterial disease management strategies for economically important crops. Finally, a brief summary of the current research that focused on the control of *X. fastidiosa* and *Candidatus* Liberibacter asiaticus is reported.

## 2. Peptides with Distinct Activity: Design and Identification of Leads

### 2.1. Antimicrobial Peptides (AMPs)

In this section, we describe the linear and cyclic peptides developed to find suitable leads as agents to control phytopathogens. With the aim of improving their biological activity profile, we have incorporated several modifications in the structure of these leads. All the research in this regard is described herein.

#### 2.1.1. Linear Peptides

**Figure 1:**
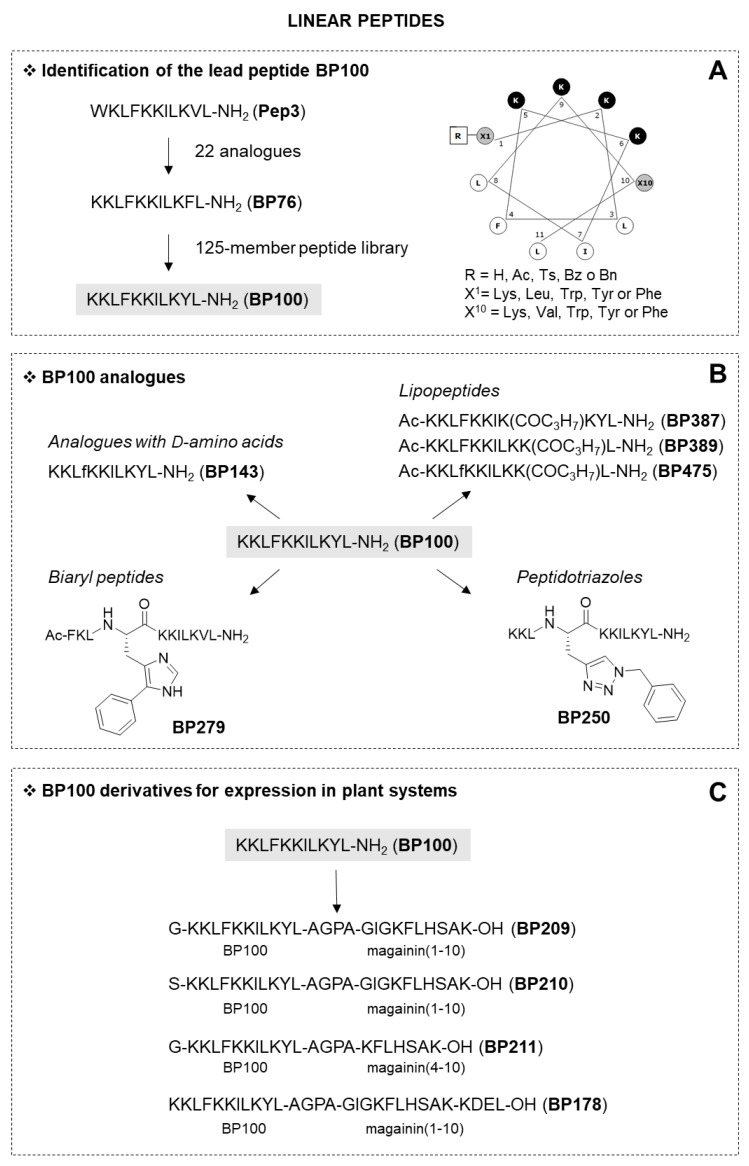
Linear peptides. Identification of the lead peptide **BP100** (**A**) [36,37], design of analogues (**B**) [39,40,41,42,43,44], and derivatives for expression in plant systems (**C**) [46], as described in Section 2.1.

The linear peptides were first designed based on the structure of the 11-residue peptide **Pep3** (WKLFKKILKVL-NH_2_), derived from the hybrid peptide cecropin A(1-7)-melittin (2-9), which was reported to display antimicrobial activity against several phytopathogens [34,35]. Taking into account the structure of **Pep3**, 22 analogues were designed by reducing its length, changing the C-terminal amide group with a carboxylic acid, derivatizing the N-terminus and replacing the residues at positions 1 and 10 (Figure 1A, Table 1). The best peptide **BP76** resulted from the replacement of Trp^1^ and Val^10^ in **Pep3** with Lys and Phe, respectively, and showed high activity against Ea, Xav and Pss (MIC values between 2.5 and 5.0 μM) and low hemolysis (34% at 150 μM) [36]. The ideal Edmunson wheel projection of **BP76** was the basis for the design of a 125-member peptide library (CECMEL11) by incorporating amino acids with various degrees of hydrophobicity and hydrophilicity at positions 1 and 10 and also by varying the N-terminal derivatization [37]. This library allowed the identification of **BP100** (KKLFKKILKYL-NH_2_), which, apart from having a good biological activity profile in vitro (MIC values between 2.5 and 7.5 μM, 22% hemolysis at 150 μM), was also more effective than **BP76** in vivo to treat Ea infections in detached apple and pear flowers and, interestingly, showed low oral acute toxicity in mice (LD_50_ > 2000 mg/kg of body weight) [38].

With the aim of further improving the biological activity of **BP100**, four sets of analogues were designed by introducing different unnatural amino acids (D-, triazolyl or biaryl amino acids) or an acyl chain in its sequence (Figure 1B, Table 1) [39]. First, 31 peptides, containing 1 to 11 D-amino acids, were prepared [40]. The incorporation of a D-amino acid is a widely used strategy to increase proteolytic stability and decrease hemolysis, while maintaining the antimicrobial activity. From this set, highlighted **BP143** (KKLf^4^KKILKYL-NH_2_) with a D-Phe^4^ (MIC values between 2.5 and 7.5 μM, 4% hemolysis at 150 μM), which displayed high activity in planta assays, and it was as effective as streptomycin for the control of bacterial blight of pepper and pear, and fire blight of pear.

A second set of **BP100** analogues incorporated a 1,2,3-triazole ring (Figure 1B, Table 1). Eleven of these analogues were designed by derivatizing the side chain of each Lys in **BP100** with a triazolyl moiety and three derivatives were obtained by replacing the benzene ring of Phe^4^ with this heterocycle [41]. It is well-known that this ring is stable to hydrolysis and redox conditions, as well as to metabolic degradation. In addition, the introduction of this heterocycle into a peptidic chain is straightforward. Peptidotriazoles with significant activity were identified. In particular, **BP250**, resulting from the replacement of the benzene ring of Phe^4^ with a benzyl triazole, displayed high antibacterial activity (MIC values between 1.6 and 12.5 μM) and no hemolysis (0% at 150 μM). The lower hemolytic activity of **BP250** compared to **BP100** could be attributed to the lower hydrophobic character of the triazole moiety compared to the benzene ring.

A third study, focused on the derivatives from the CECMEL11 library bearing a His or a 5-arylhistidine residue instead of Phe^4^, showed that this modification significantly reduced the hemolysis due to the hydrophilic character of the imidazole ring (Figure 1B, Table 1) [42]. Thus, **BP275** and **BP279** that incorporated a His^4^ or a His(5-Ph)^4^, respectively, were similarly active compared to the corresponding CECMEL11 derivatives and were considerably less hemolytic.

The fourth set consisted of lipopeptides derived from **BP100** by acylating its N-terminus or by incorporating an acyl lysine residue at each position of this sequence [43]. Butanoyl, hexanoyl and lauroyl groups were selected as acyl chains. The introduction of a fatty acid into an antimicrobial peptide has been demonstrated to improve its biological activity because it facilitates the insertion into the membrane bilayer. The best lipopeptides were **BP387** (MIC values between 1.6 and 6.2 μM, 11% hemolysis at 150 μM) and **BP389** (MIC values between 0.8 and 12.5 μM, 16% hemolysis at 150 μM), which contain a butanoyl group at position 8 and 10, respectively. Interestingly, these sequences were more active than **BP100** against the *Xanthomonas* species. From this set, 18 lipopeptides were chosen to reduce their hemolysis by incorporating a D-amino acid at position 4 [44]. This study led to the identification of **BP475** (MIC values between 1.6 and 6.2 μM, 0% hemolysis at 150 μM), bearing a D-Phe^4^ and a butanoyl group at position 10. Structural characterization of this lipopeptide by NMR revealed that it adopts an α-helix from residues 6 to 10, pointing out that the presence of D-Phe^4^ disrupts this secondary structure. Similar to **BP100 [45]**, this C-terminal α-helix may favor the insertion of this lipopeptide into the bacterial membrane bilayer, enhancing the activity.

All the above peptides were synthesized following solid-phase procedures, which is a very convenient methodology for the preparation of short peptide sequences. However, the production of peptides can also be achieved through biotechnology techniques using bacteria, yeasts or plants as biofactories. Towards this aim, peptides derived from **BP100** were designed based on the structural requirements for their expression in rice plants and to facilitate the downstream process (Figure 1C, Table 1). Thus, to achieve the minimum expressability length, **BP100** was conjugated to other units of **BP100** and to fragments of natural antimicrobial peptides, such as cecropin A, magainin and melittin [46]. One or two AGPA hinges were incorporated as a stabilitzation/distortion moiety. In addition, a signal for peptide retention in the endoplasmic reticulum (KDEL), a residue that acts as a protease recognition site (Ser or Gly) and an epitope tag for peptide detection or purification (tag54) were included. It was observed that the presence of a KDEL unit or of tag54 did not affect the biological activity. The best peptide conjugates were **BP209**, **BP210** and **BP211** (MIC values between <1.25 and 5.0 μM; 1–17% hemolysis at 150 μM), resulting from the conjugation of **BP100** to a magainin fragment through an AGPA hinge. Among the peptides including the KDEL retention signal sequence, **BP178** that incorporated **BP100** and magainin (1-10) was the more active against Ea, Xav and Pss, being also low hemolytic, and it was chosen for its expression in plants. The *BP178* gene was efficiently expressed in transgenic rice seed endosperm, where the peptide stably accumulated at 21 µg/g of seed. Interestingly, the seedlings of transgenic lines showed enhanced protection to bacterial phytopathogens [47].

#### 2.1.2. Cyclic Peptides

**Figure 2:**
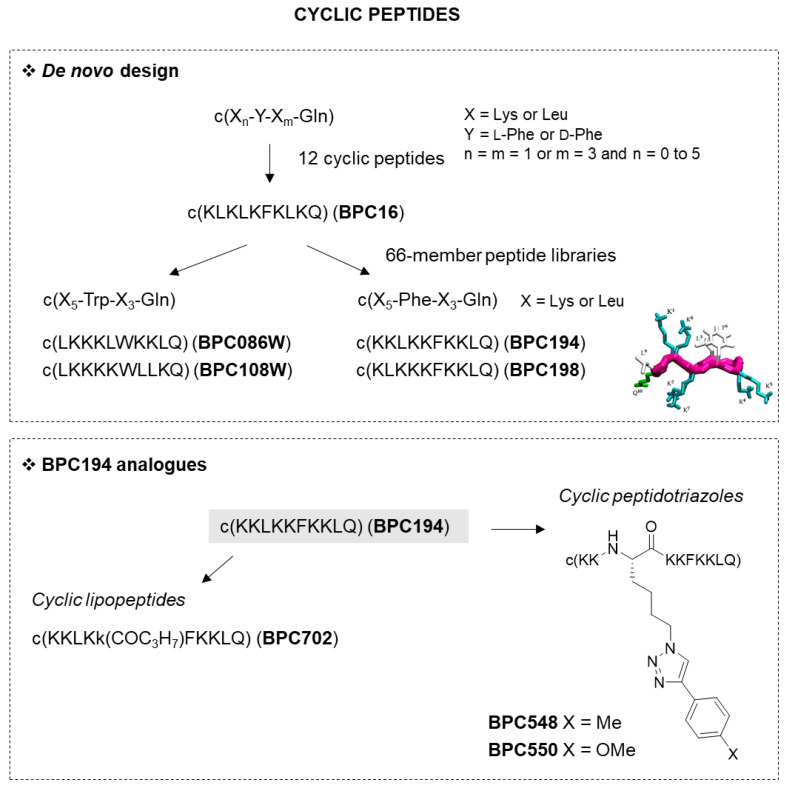
Cyclic peptides. De novo design of cyclic peptides [48,49,50,51], identification of leads and design of **BP194** analogues [49,52,53,54,55], as described in Section 2.1.

Cyclic peptides were de novo designed based on the general features described for antimicrobial peptides. Thus, cyclic peptides that contained 4 to 10 residues and incorporated hydrophilic (Lys) and hydrophobic (Leu, Phe and d-Phe) amino acids at alternating positions were prepared (Figure 2, Table 1). In addition, a Gln was incorporated at position 10 to facilitate the solid-phase synthesis. The general formula of these peptides was c(X_n_-Y-X_m_-Gln), where X was Lys or Leu, Y was l-Phe or d-Phe, m = n = 1 or m = 3 and n = 0 to 5 [48]. The most active cyclic peptide was **BPC16** (c(KLKLKFKLKQ)), which displayed MIC values between 6.2 and 12.5 μM against Pss and Xav and it was not active against Ea.

To improve the biological activity of BPC16, two combinatorial libraries were designed (Figure 2, Table 1) [49]. The first one comprised 56 cyclic decapeptides and contained Phe and Gln at positions 6 and 10, respectively, and all possible combinations of five Lys and three Leu at the other positions. From this library, the first cyclic peptides reported with activity against Ea were identified (MIC values between 12.5 and 25 μM). The second library of 16 sequences obeyed the general structure c(X^1^XXX^4^LysPheLysLysLeuGln), where X was Lys or Leu. From this library, the peptides BPC194 (c(KKLKKFKKLQ)) and BPC198 (c(KLKKKFKKLQ)) stood out for their good biological activity. BPC194 was more active against Ea (MIC values between 6.2 and 12.5 μM) than the best cyclic peptides from the first library and, in addition, both peptides were poorly hemolytic (10–13% at 150 μM). In a later study, Phe^6^ in these libraries was replaced by a Trp. The results showed that, in general, the sequences obtained were more active against Xav, Pss and Ea than the ones with Phe and showed similar hemolysis [50]. This study allowed the identification of cyclic peptides BPC086W (c(LKKKLWKKLQ)) and BPC108W (c(LKKKKWLLKQ)), with MIC values between 0.8 and 12.5 μM against these bacteria and hemolysis ≤8% at 125 μM. The active conformation of BPC194 and BPC198 was predicted by molecular dynamics simulations [51]. These conformations were the basis for the design of 15 new analogues. The best three sequences were as active as the parent peptides. Moreover, the orientation of the hydrophilic pair interactions in the active conformation of these three peptides was analogous to that in BPC194 and BPC198, but the position of the hydrophobic residues was different.

From the lead peptide BPC194, two sets of analogues were designed, bearing a 1,2,3-triazole ring and/or an acyl chain (Figure 2B, Table 1). The triazolyl derivatives resulted from replacing Leu^3^ with Ala, Glu, Lys or a Nle that incorporated a 1,2,3-triazolyl substituent at the side chain. Except for the Glu derivative, the other modifications were also included at position 5 in BPC194 instead of Lys [52,53]. This work led to cyclic peptidotriazoles BPC548 and BPC550, bearing a substituted triazolylnorleucine at position 3, with high antibacterial activity (MIC values between 3.1 and 25 μM) and low toxicity (7–12% at 150 μM).

The cyclic lipopeptides derived from BPC194 contained a fatty acid group at the *N*^ε^-amino group of a Lys residue (Figure 2B, Table 1) [54,55]. The length and the position of the acyl chain was evaluated, as well as the incorporation of one or two D-amino acids and of a His. In particular, different Lys residues in BPC194 were derivatized with acyl groups of different lengths, ranging from 4 to 18 carbon atoms. In addition, Phe^6^ or the acylated Lys were replaced with the corresponding D-enantiomer. Phe^6^ was also replaced with a His. From the 51 cyclic lipopeptides prepared, those with the highest activity contained the acyl group at Lys^1^, Lys^2^ or Lys^5^. It was observed that the presence of a D-amino acid maintained the activity and reduced the hemolysis. The incorporation of a His also resulted in a decrease in the hemolytic activity. Among the most active sequences, BPC702 was highlighted. This cyclic lipopeptide incorporated D-Lys^5^ acylated with a butanoyl group, and displayed MIC values < 12.5 μM against Xav and Pss and low hemolysis (1% at 150 μM).

**Table 1:** Antimicrobial activity (minimum inhibitory concentration, MIC) and hemolysis of selected linear and cyclic peptides.

Peptide	Sequence ^a^	MIC Intervals (μM)			Hemolysis (%) ^c^
		Ea ^b^	Pss ^b^	Xav ^b^	
*Linear*					
**BP76**	KKLFKKILKFL-NH_2_	2.5–5.0	2.5–5.0	2.5–5.0	34
**BP100**	KKLFKKILKYL-NH_2_	2.5–5.0	2.5–5.0	5.0–7.5	22
**BP143**	KKLfKKILKYL-NH_2_	2.5–5.0	2.5–5.0	5.0–7.5	4
**BP250**	KKLA(Tr-Bn)KKILKYL-NH_2_	3.1–6.2	1.6–3.1	6.2–12.5	0
**BP275**	Ts-FKLHKKILKVL-NH_2_	12.5–25	3.1–6.2	3.1–6.2	4
**BP279**	Ac-FKLH(5-Ph)KKILKVL-NH_2_	12.5–25	12.5–25	3.1–6.2	25
**BP387**	Ac-KKLFKKIK(COC_3_H_7_)KYL-NH_2_	3.1–6.2	3.1–6.2	1.6–3.1	11
**BP389**	Ac-KKLFKKILKK(COC_3_H_7_)L-NH_2_	3.1–6.2	6.2–12.5	0.8–1.6	16
**BP475**	Ac-KKLfKKILKK(COC_3_H_7_)L-NH_2_	3.1–6.2	3.1–6.2	1.6–3.1	0
**BP209**	G-KKLFKKILKYL-AGPA-GIGKFLHSAK-OH	1.2–2.5	2.5–5	<1.2	13
**BP210**	S-KKLFKKILKYL-AGPA-GIGKFLHSAK-OH	1.2–2.5	2.5–5	<1.2	17
**BP211**	G-KKLFKKILKYL-AGPA-KFLHSAK-OH	1.2–2.5	2.5–5	<1.2	1
**BP178**	KKLFKKILKYL-AGPA-GIGKFLHSAK-KDEL-OH	2.5–5.0	2.5–5.0	2.5–5.0	3
*Cyclic*					
**BPC16**	c(KLKLKFKLKQ)	>100	12.5–25	6.2–12.5	17
**BPC194**	c(KKLKKFKKLQ)	6.2–12.5	3.1–6.2	3.1–6.2	13
**BPC198**	c(KLKKKFKKLQ)	12.5–25	3.1–6.2	3.1–6.2	10
**BPC086W**	c(LKKKLWKKLQ)	6.2–12.5	3.1–6.2	0.8–1.6	8
**BPC108W**	c(LKKKKWLLKQ)	6.2–12.5	1.6–3.1	1.6–3.1	4
**BPC548**	c(KK-Nle(Tr-Ph-Me)-KKFKKLQ)	12.5–25	3.1–6.2	3.1–6.2	12
**BPC550**	c(KK-Nle(Tr-Ph-OMe)-KKFKKLQ)	12.5–25	3.1–6.2	3.1–6.2	7
**BPC702**	c(KKLKk(COC_3_H_7_)FKKLQ)	25–50	6.2–12.5	6.2–12.5	1

^a^ Lower case letters correspond to D-amino acids. Tr-Bn, triazole bearing a benzyl group. Ts, tosyl. Ac, acetyl. Ph, phenyl. COC_3_H_7_, butanoyl. Nle(Tr-Ph-Me), norleucine residue incorporating a triazole ring with a tolyl group at the side chain. Nle(Tr-Ph-OMe), norleucine residue incorporating a triazole ring with an anisole group at the side chain. ^b^ Ea, Xav and Pss stand for *Erwinia amylovora; Xanthomonas axonopodis* pv. vesicatoria and *Pseudomonas syringae* pv. syringae, respectively. ^c^ Percent hemolysis at 150 μM.

### 2.2. Plant Defense Elicitor Peptides

Antimicrobial peptides not only work directly against pathogens, but they can also act as plant defense elicitors and inducers of resistance to infections (IR). Two types of IR have been described in plants, including one that is basically pathogen-induced (systemic resistance, SAR) and another one that is caused by the colonization of plant roots by beneficial microorganisms (systemically induced resistance, ISR). This IR can be induced by molecular patterns and other molecules, including peptides [56].

With the aim of finding antimicrobial peptides that are able to trigger plant defense responses, we undertook several studies in which the following peptide elicitors were used as positive controls: **PIP-1** (YGIHTH-NH_2_) [57], **Pep-13** (VWNQPVRGFKVYE-OK) [58] and **flg15** (RINSAKDDAAGLQIA-OH), which corresponds to 15 amino acids of the N-terminal conserved domain of the well-known bacterial flagellin [59,60]. The sequences tested for their capacity to induce plant defenses included cyclic peptides, linear peptides from the CECMEL11 library, lipopeptides derived from **BP100**, cyclic lipopeptides derived from **BPC194**, linear and cyclic peptidotriazoles, as well as peptide conjugates derived from **BP100** [10,43,61,62]. The best results were obtained for the linear peptide **BP13**, the cyclic peptide **BPC200W**, the linear lipopeptide **BP378**, and the peptide conjugates **flg15-BP16** and **BP178** (Table 2). Notably, these two peptide conjugates also display high antibacterial activity; therefore, they can be considered as bifunctional compounds.

### 2.3. Bifunctional Peptides

The interesting biological activity profiles exhibited by the above peptide conjugates **flg15-BP16** and **BP178** prompted us to further explore this type of compounds. In fact, the conjugation of two antimicrobial peptides in a single sequence provides new peptides with improved activity compared to the individual monomers. However, reports on peptide conjugates, resulting from the combination of two monomers with distinct activity, are scarce. Within this context, we decided to design peptide conjugates that contained an antimicrobial peptide (**BP16**, **BP100**, **BP143**, **KSLW**, **BP387**, **BP475**) at the N- or C-terminus of a plant defense elicitor peptide (**flg15**, **BP13**, **Pep13**, **PIP-1**) [62,63]. Analysis of the in vitro and in planta activity provided two bifunctional peptide conjugates, **flg15-BP475** and **flg15-BP16**, that were able to reduce Ea infections in pear plants and Xav infections in tomato, respectively, through a dual mechanism (Table 2). On the one hand, they showed high antibacterial activity and, on the other hand, they triggered plant defense responses.

## 3. Synthesis of Peptides

Linear peptides were synthesized following solid-phase procedures using the standard 9-fluorenylmethoxycarbonyl (Fmoc)/*tert*-butyl (*t*Bu) protocol. Cyclic peptides were prepared following the orthogonal tridimensional Fmoc/*t*Bu/allyl strategy. Fmoc-Rink-MBHA or ChemMatrix resins were used as solid support. The corresponding amino acids were anchored to the solid support using common coupling reagents, such as *N,N*-diisopropyl carbodiimide (DIC) and ethyl 2-cyano-2-(hydroxyamino)acetate (Oxyma). The Fmoc group was removed by treatment with piperidine-DMF and the allyl group with Pd(PPh_3_)_4_. Cyclization was carried out with the peptide sequence linked to the solid support using [ethylcyano(hydroxyimino)acetato-*O*^2^]tri-1-pyrrolidinylphosphonium hexafluorophosphate (PyOxim)/Oxyma/*N,N*-diisopropylethylamine (DIPEA).

The peptides that incorporated a 1,2,3-triazole ring were synthesized through the reaction of an alkynyl peptidyl resin with an azide or by treatment of an azido peptidyl resin with an alkyne. The linear biaryl peptides were prepared via a Suzuki–Miyaura reaction by treating a bromopeptidyl resin with a boronic acid in solution. Alternatively, the biaryl bond was formed through the reaction of a boronopeptidyl resin, prepared by borylation of the corresponding bromopeptidyl resin, with an aryl iodide or bromide in solution.

The preparation of lipopeptides involved the synthesis of a sequence that incorporated a lysine residue with the side chain protected with 1-(4,4-dimethyl-2,6-dioxocyclohex-1-ylidene)-3-methylbutyl (ivDde). This group can be selectively removed in mild conditions that neither affect the other protecting groups nor the linkage of the sequence with the solid support. The acyl chain was incorporated using the same conditions as those of the amino acid coupling.

Once the synthesis of the sequence was completed, peptides were cleaved from the support, purified on a Combi*Flash* Rf200 automated flash chromatography system using a Redi*Sep* Rf Gold reversed-phase column packed with high-performance C_18_ derivatized silica (Teledyne ISCO, Lincoln, NE, USA). They were analyzed under standard analytical HPLC conditions with a 1260 Infinity II liquid chromatography instrument (Agilent, Santa Clara, CA, USA), using a Kromasil 100 C_18_ (4.6 mm × 40 mm, 3 µm) column. Peptides were characterized by electrospray-ionization mass spectrometry (ESI-MS) with an Esquire 6000 ESI ion trap LC/MS instrument (Bruker Daltonics, Billerica, MA, USA), equipped with an electrospray ion source. The instrument was operated in the positive ESI(+) ion mode. High-resolution mass spectrometry (HRMS) data were recorded on a Bruker MicroTof-QIITM instrument (Bruker Daltonics, Billerica, MA, USA), using ESI ionization sources. The instrument was also operated in the positive ion mode.

## 4. Activity of Peptides against Plant Pathogenic Bacteria

The use of peptides as control methods against plant pathogenic bacteria is mainly focused on the direct effect of peptides against the pathogens. Nevertheless, nowadays, there is evidence that some peptides can act in an indirect manner through the induction of the plant defense response. More information can be found in previous reviews [11,56]. Our research has taken into account both approaches, including the antibacterial activity and the ability to elicit the plant defense response.

### 4.1. In Vitro Activity

**Figure 3:**
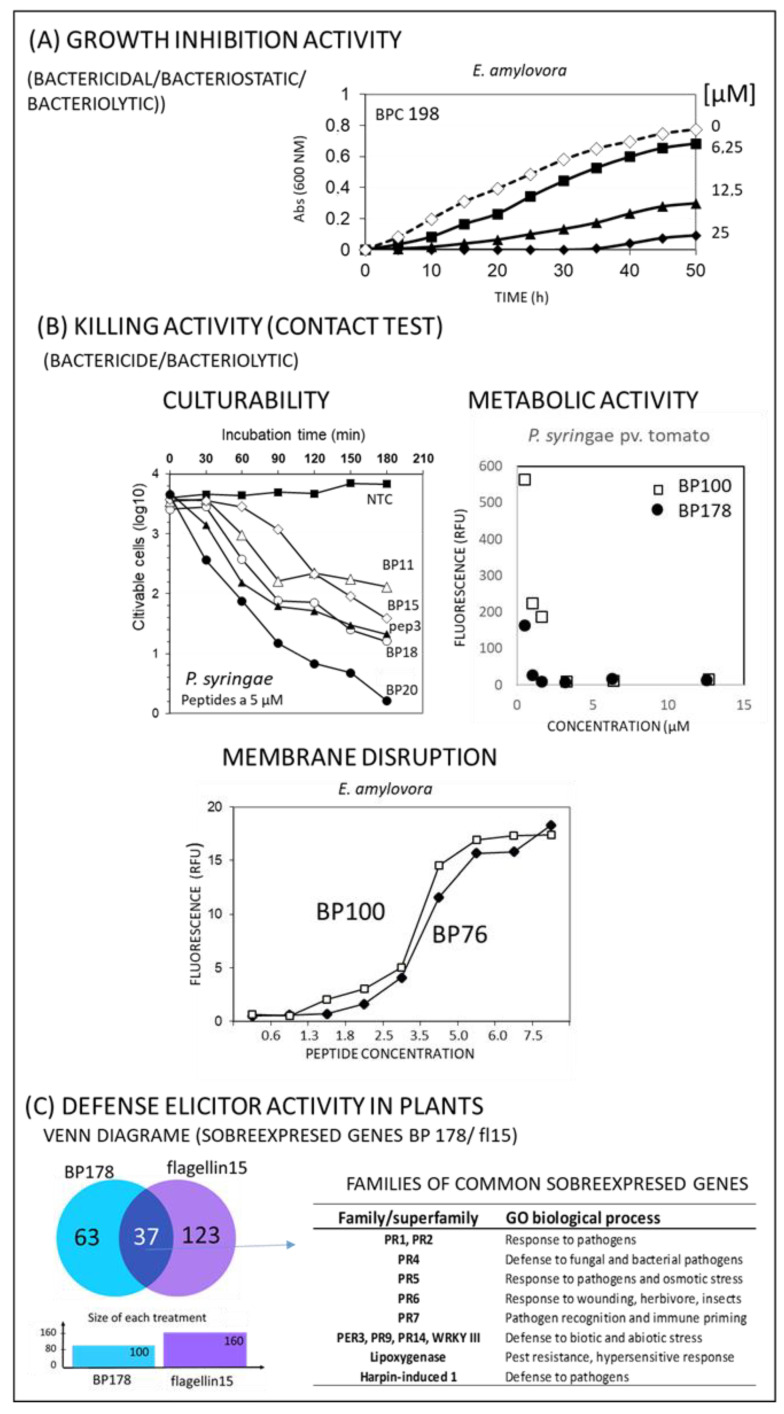
Examples of the results obtained for different peptides to determine their antibacterial activity and their defense elicitor activity in plants. (**A**) Results based on growth inhibition experiments (data from Monroc et al. [49]). (**B**) Results based on killing assays using contact tests (data from Ferre et al. and Montesinos et al. [10,36] and non-published results). (**C**) Venn diagram of the overexpressed genes in tomato plants after treatment with **BP178** and **flg15** using microarray analysis, showing 37 common overexpressed genes, and the table shows the family of common overexpressed genes and their Gene Ontology biological process (data from Montesinos et al. [10]).

To determine the in vitro antibacterial activity of the peptides, the biological characteristics of the pathogens have to be taken into account, including their ability to grow on culture plates and their nutritional needs. Almost all of the phytopathogenic bacteria that we studied grew well in nutritional broth and agar plates and did not have complex nutritional requirements. Therefore, although some of bacteria of the genus *Pseudomonas*, *Erwinia*, and *Xanthomonas* are considered quarantine phytopathogens in some zones (found in the entire EU territory but not in some countries or zones, EU 2019/2072 of 28 November 2019), conventional methods, such as viable plate counting, could be used. Depending on the type of antimicrobial activity to be assessed after exposure of the pathogens to the peptides, several methodologies could be employed, such as those that determine the metabolic activity of cells or the amount of the free DNA in the sample due to pore formation (Figure 3). The most widely used methodology is the growth inhibition test in nutritional broth by measuring the optical density (Figure 3A). However, it has to be noted that this test does not allow to determine if the peptide has bacteriostatic or bactericidal activity. To determine the killing activity, contact tests should be used, and the effect can be measured as culturable cells recovered or metabolic activity (e.g., resarzurin). The bacteriolytic activity can be determined using the dye SYTOX green, which binds to free DNA and is able to enter the cells with damaged membranes due to the formation of pores by the peptide (Figure 3B).

### 4.2. Defense Elicitor Activity of Peptides in Plants

To determine the defense elicitor activity of the peptides, we started analyzing the capacity of selected peptides to induce extracellular pH changes and hydrogen peroxide production in tobacco cell cultures that are considered indicators of eliciting activity [61]. However, an in planta screening platform was preferred, so, we developed a platform in which tomato plant was used as a model plant and peptides were applied through a spray. The expression of defense-related genes was assessed by performing a retrotranscription of the RNA extracted from the plant coupled to a quantitative PCR, using actine as the endogenous gene for the normalization of the results and the ΔΔCt method to perform the relative quantification of the treatment compared to the non-treated control [49]. Different types of peptides were tested, such as linear, cyclic and conjugates [43,61,62,64,65]. The results were promising and allowed the identification of peptide conjugates, incorporating an antimicrobial peptide and a plant defense elicitor peptide, that displayed bifunctional activity.

Taking into account the promising results of the peptide conjugates, in particular those of **BP178**, experiments were carried out taking into account the whole genome of the plants, including microarrays in tomato plants and RNAseq in *Prunus dulcis* using the Illumina platform. A microarray analysis of tomato plants (GeneChipTM tomato gene 1.0 ST array (Affymetrix)), treated with **BP178,** was performed. **flg15**, ethylene (ET), salicylic acid (SA) and jasmonic acid (JA) were used as the reference compounds [10]. It could be concluded that **BP178** elicits a similar gene expression pattern related to defenses than **flg15** and that it is related to several defense pathways (ET, SA, JA) (Figure 3C). Nevertheless, using tomato plant as a model system does not mean that the observed response will be the same in other plants of interest. Therefore, it is better to use the desired host of study whenever possible. In that sense, **BP178** was applied by endotherapy to almond plants, which were analyzed using RNAseq to obtain a holistic view of the effect of the peptide in the almond transcriptome. The results confirmed the observations obtained in tomato plants regarding the ability of **BP178** to elicit a defense response [62].

### 4.3. Other Activities

**Figure 4:**
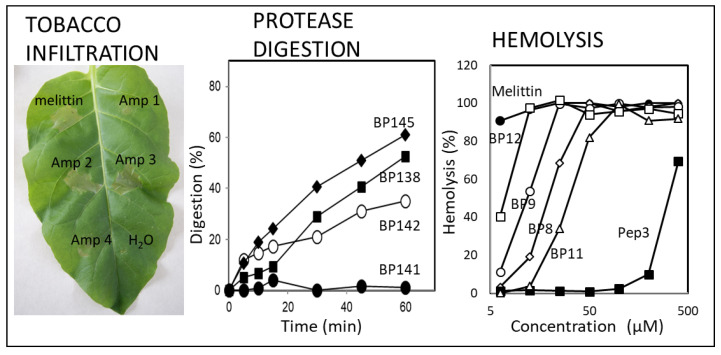
Examples of results obtained for different peptides related to phytotoxicity on tobacco leaves to the susceptibility to protease degradation using proteinase K and to the hemolytic activity using blood erythrocytes. Data from Ferre et al. and Güell et al. [36,40].

Other activities that need to be taken into account for the development of a peptide-based product are the phytotoxicity, the protease susceptibility and the hemolytic activity (Figure 4). To assess the phytotoxicity, peptides are placed into the mesophyll of tobacco plants and the diameter of the lesion they cause is measured. This assay shows the direct activity of peptides in plant cells, which must be taken into account when selecting the peptides to be tested in planta.

Another parameter to consider is the susceptibility of peptides to protease degradation, since they will be exposed to proteases once applied to the plants. If the peptides are not resistant enough, their half-life time will be low. With that in mind, the sequence of the lead peptides has been modified through the incorporation of unnatural amino acids, as mentioned above. For example, **BP143**, which bears a D-Phe^4^, displayed 18% degradation after being exposed to proteinase K for 1 h compared to its parent peptide **BP100**, which showed 75% degradation [40].

Finally, hemolytic activity is the capacity to lyse erythrocytes, which may give an idea of the peptide toxicity. Therefore, it is important for peptides to display low hemolysis, since it will indicate that they are safe for the manipulators in future field applications. Many peptide modifications have allowed us to obtain peptides with similar or higher antimicrobial activity and, at the same time, with lower hemolytic activity. This is the case of **BP143**, in which the incorporation of a D-Phe^4^ in **BP100** led to a reduction in the rate of hemolysis from 54% to 5% [40].

The results of tobacco leaf infiltration and the hemolysis assays are indicative of the potential toxicity of peptides. However, toxicity tests on whole plants or animals have to be performed before further development stages, because the main method of interaction of peptides with humans and animals will be dermal, by inhalation or oral ingestion. Therefore, it is essential to know the acute toxicity of these compounds. Based on this, peptides **BP100**, **BP76** and **BP15** were tested for oral acute toxicity in mice, with the median lethal dose (LD_50_) and the lower limit lethal dose (LLD) being higher than 1000–2000 mg/Kg of body weight, which is considered a very low oral toxicity [66].

## 5. Control of Infections in Plant Material Caused by Phytopathogenic Bacteria in Controlled Environment Conditions

To assess the activity of peptides in vegetal material in controlled environment conditions, two strategies were performed. One strategy consists of using detached vegetal material, such as leaves, flowers or immature fruits and the other consists of using the whole plant under greenhouse conditions.

For example, while developing analogues of **BP100** that contained a D-amino acid in their sequence, a first experiment was performed with all the analogues using pear tree leaves for Pss, pepper leaves for Xav and pear immature fruits for Ea. This experiment allowed us to select the analogues **BP143** and **BP145**. Afterwards, these peptides were applied to whole plants using the same pathosystems, which demonstrated that **BP143** was as effective as streptomycin. Its activity was also greater than **BP100** in controlling Pss and Xav [40]. Aside from these analogues based on D-amino acid substitutions, the linear peptide **BP13** and the cyclic peptide **BPC200W** were also tested in pear trees, since they had antibacterial activity and were able to elicit the plant defense response. These peptides were applied using two different treatment schedules (two or three treatments before the inoculation of the pathogen); **BP100** and **BP143** were added to the experiment as reference peptides. The reference peptides only showed a significative reduction in the disease when three treatments were used, whereas **BP13** and **BP200W** were effective in all treatments [61].

**Figure 5:**
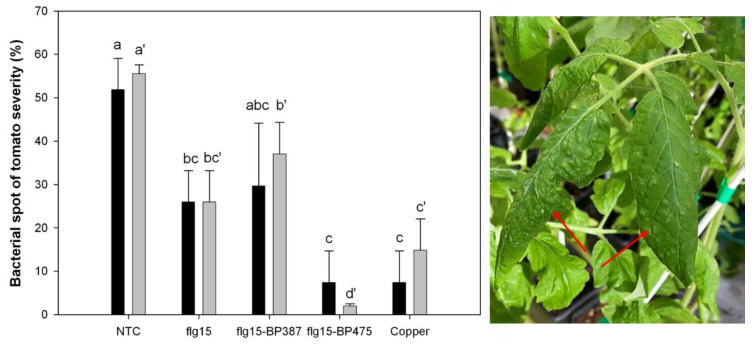
Effect of the application of **flg15** and of the peptide conjugates **flg15-BP387** and **flg15-BP475** on the disease severity of bacterial leaf spot blight caused by *X. axonopodis* pv. vesicatoria in tomato plants. Black and grey are the two independent experiments. The confidence intervals for the means are indicated on top of the bars. Different letters show significant differences between the treatments according to Duncan’s test (*p* < 0.05, ANOVA, LSD) for the two experiments performed (with and without apostrophe). Data from Caravaca-Fuentes et al. [62]. Picture shows the disease symptoms in tomato leaves (red arrows).

Regarding the bifunctional peptide conjugates, an experiment was performed with **flg15**-**BP16** to assess their ability to control Ea infections on pear plants, when applied 48 h before the inoculation of the pathogen. In this experiment, the plants were also treated with the monomers of the conjugate, **flg15** and **BP16**, independently and combined for comparative purposes. The results showed that **flg15**-**BP16** and its monomers applied together (**BP16** and **flg15**) presented similar values to those of the treatment with kasugamycin [62]. In the case of other peptide conjugates, the effect of changing the elicitor peptide (**flg15** or **PIPI**), but not modifying the antimicrobial peptide (**BP475**), and the other way around by changing the antimicrobial peptide (**BP475** or **BP387**), but keeping the elicitor peptide (**flg15**), was studied for the control of infections caused by Xav in pepper plants. Peptide conjugate **flg15-BP475** showed an efficacy similar or higher than copper oxychloride (Figure 5) [63]. All these results are summarized in Table 2. The peptide conjugate **BP178** was tested in tomato plants to control Xav and Pst infections. **BP178** was applied using a spray and the results were compared to those obtained for **BP100**. When **BP178** was applied preventively 1 day before the inoculation of the pathogen, 70% efficacy in the control of the disease severity was observed. In addition, tomato plants with **BP178** showed significantly increased expression of about 100 genes of which 74.4% were related to plant defense and, particularly, to biotic stress responses using microarray analysis (Figure 3C) [10].

**Table 2:** Peptides assayed in planta for elicitation properties (overexpresed genes in tomato) and in detached plant material or plants for disease control in different pathosystems. Data extracted from several papers cited in the main text.

Peptide	Sequence ^a^	Pathosystem	Gene Overexpression ^b^
**BP13**	FKLFKKILKVL-NH_2_	Ea/pear	3/11
**BP16**	KKLFKKILKKL-NH_2_	Ea/pear	1/11
**BP100**	KKLFKKILKYL-NH_2_	Xav/pepper, Pss/pear, Ea/pear	2/11
**BP143**	KKLfKKILKYL-NH_2_	Xav/pepper, Pss/pear, Ea/pear	2/11
**BP378**	Ac-KKLFKKILKYK(COC_5_H_11_)-NH_2_	-	3/11
**BPC200W**	c(LLLLKWKKLQ)	Ea/pear	4/11
**BP178**	KKLFKKILKYL-AGPA-GIGKFLHSAK-KDEL-OH	Pst/Xav/tomato	8/11
**flg15-BP16**	RINSAKDDAAGLQIA-KKLFKKILKKL-NH_2_	Ea/pear	6/11
**flg15-BP475**	Ac-RINSAKDDAAGLQIA-KKLfKKILKK(COC_3_H_7_)L-NH_2_	Xav/tomato	7/11

^a^ Lower case letters correspond to d-amino acids. COC_3_H_7_, butanoyl. COC_5_H_11_, hexanoyl. ^b^ Number of overexpresed genes compared to the number of genes studied.

## 6. Concluding Remarks

In summary, significant research has been carried out regarding the development of peptides as agents to control plant pathogenic bacteria of economic importance. Linear and cyclic peptides have been designed and synthesized, which have provided leads that have been further optimized by incorporating several structural modifications. This approach led to the identification of sequences with high antibacterial activity, low hemolysis and high stability to protease degradation. In addition, our research has not only focused on peptides that kill bacteria, but also on sequences with the ability to elicit plant defense responses. The achievement of these purposes has required the improvement of methods and technologies for the in vitro and/or in planta screening of a large number of compounds for their antibacterial, hemolytic and phytotoxic activities, as well as for their defense elicitor activity in plants.

The research along these lines has resulted in sequences with distinct biological activities, including antibacterial peptides, plant defense elicitor peptides and sequences with both activities. Thus, we have developed a versatile platform that can be useful at finding, with a certain guarantee of success, peptides that are able to control plant diseases caused by other bacteria. Moreover, the best peptides constitute good candidates with suitable properties to be further developed as effective phytosanitary products for their use in agriculture. Therefore, these peptides represent an alternative strategy that should be taken into account in the management of plant diseases in fields. Multidisciplinary work that involves different research areas, such as microbiology, organic chemistry and combinatorial chemistry, and phytopathology, is crucial in achieving our goal.

## 7. Ongoing Research

Nowadays, our research on the use of peptides as biopesticides is focused on the control of the xylem-restricted quarantine phytopathogen *Xylella fastidiosa* (Xf), which is the cause of several quarantine diseases found in the EU, such as the olive quick decline (OQDS), and on the control of the floem-restricted *Candidatus* Liberibacter asiaticus (CLas), causing citrus *huanglongbing* (HLB), previously called citrus greening disease, which is one of the most destructive diseases of citrus worldwide. The assessment of the antimicrobial activity of peptides against Xf and CLas is challenging because the former is a fastidious phytopathogen microorganism with a slow growth rate and special nutritional requirements, and the latter is considered a non-culturable microorganism. In the case of Xf, the previously mentioned conventional methods to assess the activity of peptides, such as the in vitro contact test, can be used but their antimicrobial activity can be overestimated [67]. Therefore, methodologies based on molecular methods had to be developed. These methods allow for the evaluation of bacterial viability, without taking into account its culturability [67,68]. Regarding CLas, researchers are using *Liberibacter crescens,* which is the closest cultured relative of this important uncultured crop pathogen.

The formation of biofilm in the xylem vessels of the host is one of the main pathogenicity mechanisms of *X. fastidiosa;* thus, we adapted a methodology using crystal violet [69] to identify peptides with antibiofilm activity against these bacteria [70]. We also focused our attention on the design of peptides that targeted the pathogenicity functions of *X. fastidiosa*. Furthermore, we are currently studying the peptides that are able to elicit defense responses in almond plants, because it is one of the most important crops in our research.

In addition, we have performed *in planta* experiments in *Prunus dulcis* Avijor inoculated with *X. fastidiosa* and treated with the bifunctional peptide **BP178** using endotherapy. The peptide was applied using a preventive and a curative strategy, in addition to a combination of both. The results showed that the combined strategy (preventive and curative) is able to reduce the population levels of *X. fastidiosa* and the disease severity [71].
